# Texas and the Great Tuberculosis Congress

**Published:** 1906-08

**Authors:** 


### Texas and The Great Tuberculosis Congress.

Texas, "The Empire of the West," has been active and in the lead in the propaganda against consumption, and the work of her medical men and journalists was promptly recognized at the St. Louis Congress in October, 1904, by the election of Texas men to the office of President and Secretary. It is an honor to our great State, and our Governor and the medical profession will show their appreciation of it by a very large representation of our strong men,

*This paper appears in last issue of the *Medico-Legal Journal* and in the *Texas Medical News* for July (ult.).

selected from every section of the State, at the coming meeting in New York. Texas will be represented as follows:

Honorary President for Texas: S. W. T. Lanham, Governor of Texas.

Honorary Vice-Presidents for Texas: Geo. R. Tabor, M. D., State Health Officer and Surgeon-General of Texas; David Franklin Houston, LL. D., President University of Texas.

President of the Congress: F. E. Daniel, M. D., Austin, Texas.

General Secretary of the Congress: M. M. Smith, M. D., Austin, Texas.

Delegates (appointed by the Governor): Marvin L. Graves, M. D., Professor of Medicine, Medical Department, University of Texas; J. W. McLaughlin, M. D., late Professor, Practice of Medicine, Medical Department University of Texas; B. M. Worsham, M. D., Superintendent State Insane Asylum, Austin; G. B. Foscue, M. D., President Texas State Medical Association; J. E. Gilcreest, M. D., ex-President Texas State Medical Association; Ira Carleton Chase, M. D., Secretary State Medical Association, and editor *State Medical Journal;* Drs. Boyd Cornick, San Angelo; M. J. Bleim, San Antonio; Malone Duggan, San Antonio; M. B. Grace, Seguin; A. E. Spohn, Corpus Christi; J. H. Florence, Dallas; C. H. Wilkinson, Galveston; R. W. Knox, Houston; S. R. Burroughs, Buffalo; J. T. Wilson, Sherman; H. W. Cummings, Hearne; Bacon Saunders, Fort Worth; W. G. Jameson, Palestine; C. A. Smith, Texarkana; W. N. Vilas, El Paso; J. A. Rawlings, El Paso; John Preston, Abilene; P. C. Coleman, Colorado City; A. S. Garrett, Springtown; D. R. Fly, Amarillo; C. M. Alexander, Coleman; B. F. Kingsley, San Antonio; Nettie Klein, Texarkana; W. W. Wilcox, Laredo; T. J. Bennett, Austin; J. C. Anderson, Granger; F. W. Kirkham, Cuero; John T. Moore, Galveston; C. W. Truehart, Galveston; D. S. Weir, Beaumont; W. B. Collins, Lovelady; E. N. Shaw, Cameron; O. I. Halbert, Waco; Albert Woldert, Tyler; J. J. Robert, Hillsboro; J. M. Frazier, Belton; S. P. Rice, Marlin; C. B. Raines, Mineral Wells; Alf. Irby, Weatherford; C. M. Rosser, Dallas; D. J. Jenkins, Daingerfield; J. C. Loggins, Ennis; M. Smith, Sulphur Springs; J. A. Daniels, Carthage.

